# Classification of lung pathologies in neonates using dual-tree complex wavelet transform

**DOI:** 10.1186/s12938-023-01184-x

**Published:** 2023-12-04

**Authors:** Sagarjit Aujla, Adel Mohamed, Ryan Tan, Karl Magtibay, Randy Tan, Lei Gao, Naimul Khan, Karthikeyan Umapathy

**Affiliations:** 1 Department of Electrical, Computer, and Biomedical Engineering, Toronto Metropolitan University, 350 Victoria Street, Toronto, ON M5B 2K3 Canada; 2 Department of Pediatrics, Mount Sinai Hospital, 600 University Ave, Toronto, ON M5G 1X5 Canada

**Keywords:** Neonatal lung ultrasound, Image analysis, Wavelet decomposition, Pattern classification

## Abstract

**Introduction:**

Undiagnosed and untreated lung pathologies are among the leading causes of neonatal deaths in developing countries. Lung Ultrasound (LUS) has been widely accepted as a diagnostic tool for neonatal lung pathologies due to its affordability, portability, and safety. However, healthcare institutions in developing countries lack well-trained clinicians to interpret LUS images, which limits the use of LUS, especially in remote areas. An automated point-of-care tool that could screen and capture LUS morphologies associated with neonatal lung pathologies could aid in rapid and accurate diagnosis.

**Methods:**

We propose a framework for classifying the six most common neonatal lung pathologies using spatially localized line and texture patterns extracted via 2D dual-tree complex wavelet transform (DTCWT). We acquired 1550 LUS images from 42 neonates with varying numbers of lung pathologies. Furthermore, we balanced our data set to avoid bias towards a pathology class.

**Results:**

Using DTCWT and clinical features as inputs to a linear discriminant analysis (LDA), our approach achieved a per-image cross-validated classification accuracy of 74.39% for the imbalanced data set. Our classification accuracy improved to 92.78% after balancing our data set. Moreover, our proposed framework achieved a maximum per-subject cross-validated classification accuracy of 64.97% with an imbalanced data set while using a balanced data set improves its classification accuracy up to 81.53%.

**Conclusion:**

Our work could aid in automating the diagnosis of lung pathologies among neonates using LUS. Rapid and accurate diagnosis of lung pathologies could help to decrease neonatal deaths in healthcare institutions that lack well-trained clinicians, especially in developing countries.

## Introduction

Respiratory diseases in newborns are the leading cause of admission to Neonatal Intensive Care Units (NICU) [1]. In recent years, lung ultrasound (LUS) has emerged as a promising and exciting application in neonatal point-of-care ultrasound (POC-US). Recent articles have demonstrated that ultrasound imaging can be just as effective, if not more so, than traditional X-rays as a diagnostic modality [2–4]. For example, LUS is easily available at bedside, provides real-time imaging and is free of radiation hazards [2, 3]. Additionally, it has been shown to be better than X-ray in diagnosing neonatal respiratory distress syndrome (RDS) [4]. However, healthcare institutions in developing countries lack well-trained clinicians to interpret LUS images expertly and diagnose neonatal lung pathologies accurately. An automated framework for classifying LUS images of typical neonatal lung pathologies could aid in rapid and accurate detection.

**Fig. 1:**
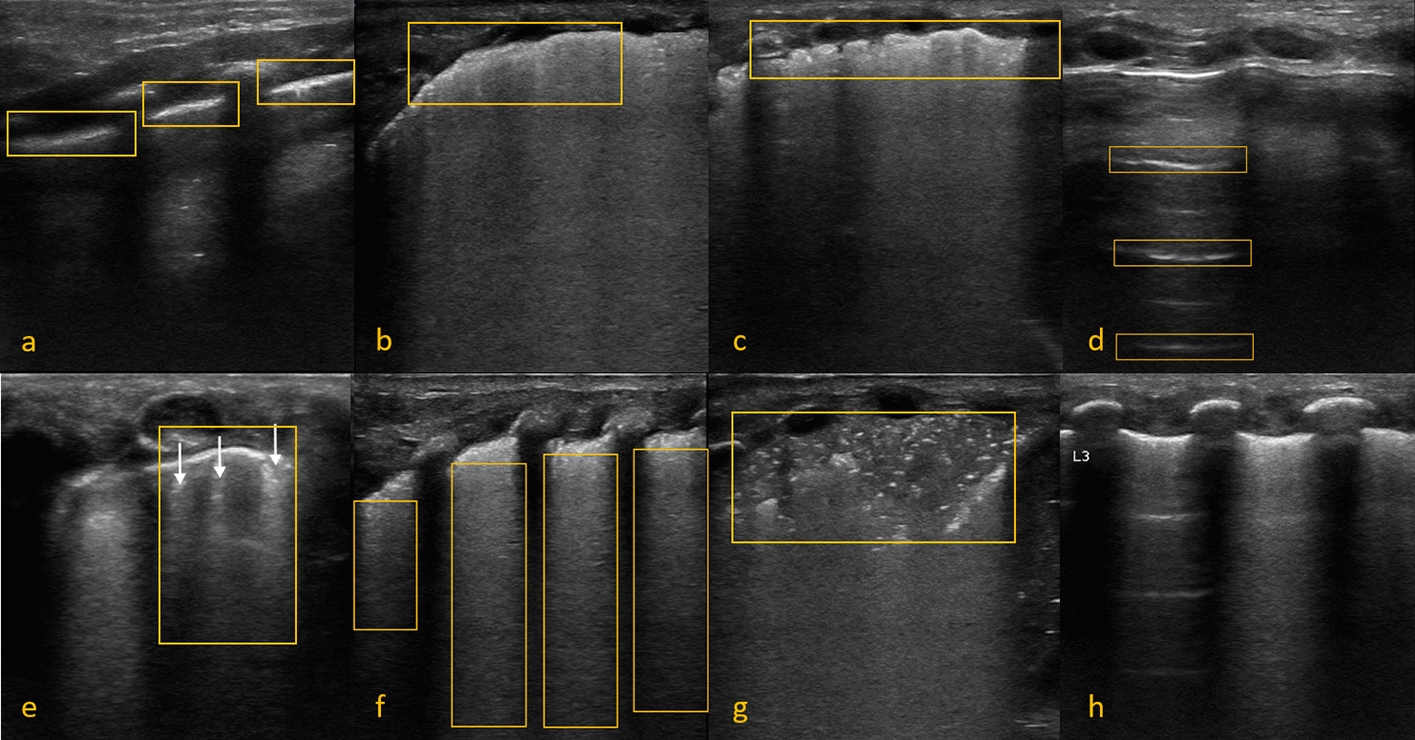
**a**: A sample normal Pleura from a subject with Normal Lung, **b** A sample thick Pleura (> 2 mm) from a subject with CON, **c** A sample thick and irregular Pleura from a subject with chronic lung disease, **d** Sample A-Lines from a subject with PTX, **e** Sample separate B-Lines from a subject with TTN, **f** Sample coalescent B-lines from a subject with RDS, **g** Sample CON illustration from a subject with Consolidation, and **h** Sample Double Lung Point from a subject with TTN

In a neonatal intensive care unit (NICU), the most common lung pathologies are transient tachypnea (TTN), pneumothorax (PTX), respiratory distress syndrome (RDS), consolidations (CON), and chronic lung disease (CLD). Clinicians characterize LUS morphologies to classify neonatal lung pathologies. Typical LUS morphologies are Pleural lines, A-lines, Separate or Coalescent B-lines, and Consolidations [5]. Pleural lines, shown in Fig. 1A, appear as a hyperechoic line that represents the junction of the visceral and the parietal pleura. A well-defined and sliding pleural line signifies a normally appearing lung and rule out PTX; Fig. 1D shows A-lines representing reverberation artifacts and appearing as horizontal, equidistant parallel lines. These lines are commonly seen in healthy individuals and may be erased (by B lines) or enhanced in the presence of PTX; B-lines are vertical line artifacts that extend from the pleural line to the bottom of an LUS image. B-lines could be either separate or coalescent. Separate B-lines (Fig. 1E) indicate thickened interlobular septa in the lungs, and its intensity is correlated with the amount of fluid inflammation present in the lungs. The presence of multiple distinct B-lines could signify interstitial lung disease. Coalescent B-lines (1F) are characteristic of alveolar-interstitial syndrome. Coalescent B-lines are formed due to the fusion of separate B-lines; consolidations is described as the presence of hypoechoic areas surrounded by hyperechoic short lines and irregular or absent pleural lines, as shown in Fig. 1G. CON are typically observed in LUS images due to a severe lack of aeration of the lungs; Finally, a combination of A-lines and B-lines artifacts may indicate “Double Lung Point” that is caused by the difference in aeration of the upper and lower lung regions (Fig. 1H). Double Lung Point is a characteristic feature of TTN, which results from fluid retained in the lungs of newborns, causing them to breathe faster and harder [6]. Furthermore, RDS has LUS morphology that appears as a combination of irregular and thickened pleural line, CON and Coalescent B-line. CLD also appears as an irregular and thickened pleural line with the addition of varying intensities of separate and coalescent B-lines. A description of the LUS morphologies is provided in Table 1 and a matching of the morphologies to LUS pathologies is given in Table 2.

**Table 1:** Descriptions of LUS morphologies

Morphology	Description
Pleural line	Hyperechoic line that represents the junction of the visceral and the parietal pleura.
A-lines	Represent reverberation artifacts and appear a horizontal, equidistant parallel lines.
Separate B-lines	B-lines are vertical line artifacts that extend from the pleural line to the bottom of an LUS image. Separate B-lines indicate thickened interlobular septa in the lungs, and their intensity is correlated with the amount of fluid inflammation present in the lungs. The presence of multiple distinct B-lines could signify interstitial lung disease.
Coalescent B-lines	Coalescent B-lines are characteristic of alveolar-interstitial syndrome. Coalescent B-lines form due to the fusion of separate B-lines.
Consolidation	The presence of hypoechoic areas surrounded by hyperechoic short line and irregular or absent pleural lines. Indicates areas of pneumonia or atelectasis.
Lung sliding	The back-and-forth sliding of the pleural line with respiration.

**Table 2:** LUS morphologies of LUS pathologies

	Normal	TTN	PTX	RDS	CLD	CON
A-lines	X	X	X			
Normal pleural line	X	X				
Thick pleural line				X	X	X
Irregular pleural line				X	X	X
Lung sliding	X	X		X	X	X
Separate B-lines		X		X	X	X
Coalescent B-lines		X		X	X	X
Consolidation				X	X	X

Previous works have attempted to automate the detection of lung pathologies using LUS morphologies. Summers et al. developed a diagnostic assistant to detect LUS morphologies related to PTX among adults [7]. A prenatal study by Jiao et al. predicted the likelihood of neonatal respiratory morbidity (NRM) based on spatial and textural LUS image features, gestational age (GA), and gestational diabetes mellitus (GDM) [8]. Similarly, Bonet et al. [9] showed that indicators of RDS and CLD in neonates could be automatically detected from fetal LUS images using combinations of textural features and GA. Du et al. also used texture, clinical features, and spatiotemporal features to categorize LUS images with a higher likelihood of NRM [10, 11]. Veeramani et al. automated classification of PTX, RDS, and TTN from LUS images, among other lung pathologies, using over six hundred features among which are textural patterns typical of neonatal LUS morphologies [12].

While the aforementioned works automated the classification of lung pathologies from primarily fetal LUS images, POC-US studies for diagnosing lung pathologies in neonates using LUS morphologies are lacking in the current literature. Moreover, previous studies examined only a subset of the most common neonatal lung pathologies. Thus, our current work aims to automate the detection and classification of lung pathologies in neonates using textural and morphological features of LUS images. Our current work also aims to expand the classification for all neonatal lung pathologies commonly encountered by clinicians in a NICU. Our work could aid in improving the diagnosis of neonatal lung pathologies and alleviate neonatal mortality in developing countries needing well-trained physicians.

Our previous works attempt to contribute to automating the detection and classification of common neonatal lung pathologies. In Bassiouny et al. [13], seven important LUS morphologies for specific lung conditions were identified using a Faster Region-Based Convolutional Neural Network (FRCNN) object detection model. Moreover, we previously used recurrence analysis to automatically detect recurrent non-linear LUS image features of common neonatal lung pathologies, such as A- and B-lines [14]. With an expanded data set, we extend our initial work by isolating and extracting localized LUS line and texture patterns of the most common neonatal lung diseases using a two-dimensional (2D) Dual-Tree Complex Wavelet Transform (DTCWT) [15]. Since wavelets have been shown to effectively capture spatially and temporally varying features [16, 17], wavelets may also be able to capture LUS morphologies. The works by Cao et al. [18] and Amin et al. [19] demonstrated wavelet features associated with ultrasound image morphologies characteristic of COVID and fatty liver, respectively. Their proposed wavelet features could also be useful for detecting neonatal lung pathologies from LUS images.

**Fig. 2:**
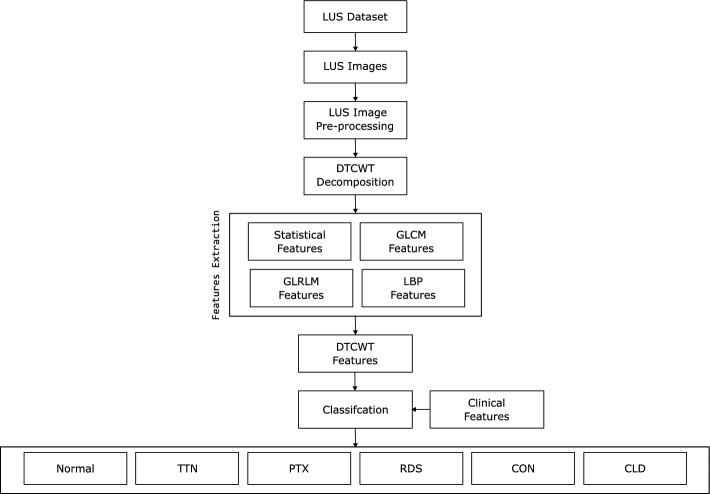
A block diagram of our proposed image feature extraction and classification framework

DTCWT is effective in extracting localized spatiotemporal features from signals and images. Chen et al. previously used 1D DTCWT to extract electroencephalography (EEG) signal features for seizure detection [20]. Aydogan et al. also proposed using 2D DTCWT on Magnetic Resonance (MR) images to detect bone fractures and segment and classify brain tumors [21]. 2D DTCWT has also been used to extract spatiotemporal local features of ultrasound images of the thyroid glands to classify organ-specific diseases [22]. Therefore, we could use 2D DTCWT to extract features from LUS images of neonates and hone in on localized features indicative of specific lung pathologies. In Fig. 2, we show a block diagram of our proposed framework to automate the detection of LUS morphologies.

The key research contributions of the paper are listed below:

- Extracted line and texture features from the DTCWT decomposition of neonatal LUS images.
- Utilized the DTCWT and clinical features to classify typical LUS pathologies among neonates using a Linear Discriminant Analysis model.
- Models were tested using 2 data sets, a balanced and an imbalanced data set, as well as 2 cross-validation methods: leave-one-out cross-validation (LOO CV) and leave-one-subject-out cross-validation (LOSO CV).
- The proposed research can help assist the clinical community, especially in remote hospitals, in screening for neonatal lung conditions and ensuring early identification of potential issues.

Our work is organized as follows. We present our results in Section 2. We discuss how our results relate to the literature on LUS morphology classification in Section 3. In Section 4, we present our conclusions and the implications of our findings to the rapid and accurate diagnosis of neonatal lung pathologies. We describe in Section 5 our data set, image preprocessing and decomposition, feature extraction, and classification methods. Finally, in Section 6 we provide our declarations including a list of all abbreviations.

## Results

We performed the following four 6-group classification experiments: **(i)** Linear Discriminant Analysis (LDA) with Leave-one-out Cross Validation (LOO CV) on a balanced data set, **(ii)** LDA with LOO CV on the imbalanced data set, **(iii)** LDA with Leave-One-Subject-Out Cross Validation (LOSO CV) on a balanced data set and **(iv)** LDA and LOSO CV on the imbalanced data set. In performing the above experiments, we also restricted the feature dimension to the top 15 DTCWT features selected by the feature selection method and three clinical features. Considering the approximate training group sample sizes, 18 features will form  10–15% of the training group sizes, which is expected to produce conservative performance without over-fitting. We also tested the distribution of top-selected features by the feature selection method. We found that, in general, Grey-Level Co-occurrence Matrix (GLCM) and Grey-Level Run Length Matrix (GLRLM) features were selected in larger proportions than statistical and Linear Binary Pattern (LBP) features.

**Fig. 3:**
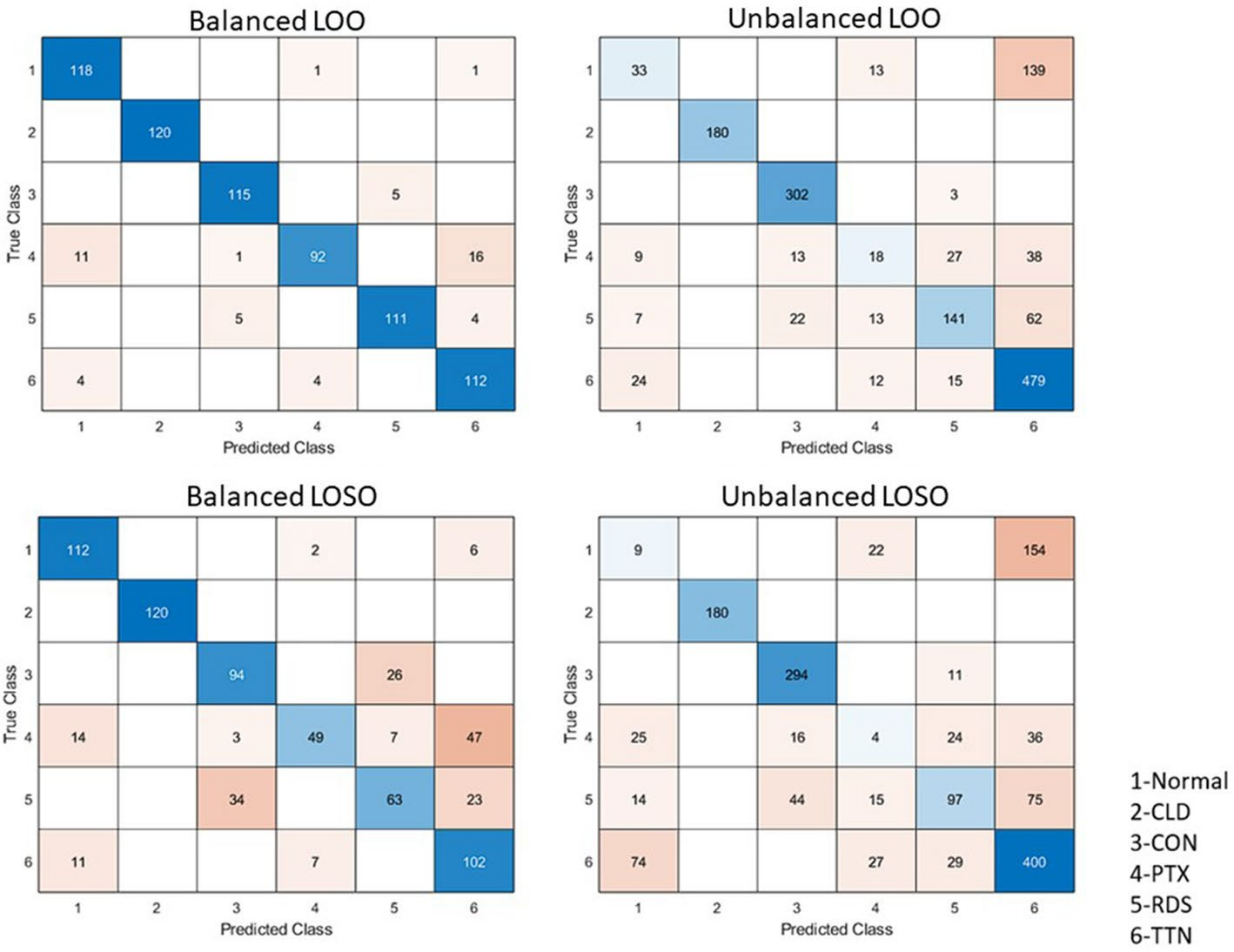
Confusion matrices for all results

The obtained results are presented in the four confusion matrices in Tables 3, 4, 5 and 6. Table 3 presents the results of the 6-group classification with LOO CV on the balanced data set. With an overall per-image classification accuracy of **92.78%**, most groups are classified well except PTX, which slightly overlaps between Normal and TTN. Table 4 presents the results of the 6-group classification with LOO CV on the imbalanced data set. With an overall per-image classification accuracy of **74.39%**, the performance drops considerably compared to the balanced data set. As evident from Table 4, looking at the TTN column, most of the issue seems to overlap with TTN. This is expected as the data set is skewed due to almost 1/3rd of TTN cases. As confirmed by our clinical collaborator, TTN can have a varying and overlapping presentation with Normal, PTX, and RDS. Specifically, for the imbalanced data set experiment, Normal was misclassified as TNN 75.14% of the time, PTX was misclassified as TTN 36.19% of the time, and RDS was misclassified as TTN 25.31% of the time. These three conditions look very similar. They all commonly have A-lines. However, there are differences between these diseases, such as the amount of fluid/air ratio present in the lungs, which can be picked up in the balanced data set. These might have contributed to the reduced performance of the per-image classification using the imbalanced data set. The weighted F1 score was 0.927 on the balanced data set and 0.740 on the imbalanced data set. Confusion matrices for the results are provided in Fig. 3.

**Table 3:** Classification confusion matrix for results with LOO CV on the BALANCED data set using top 15 DTCWT features and three clinical features. The overall per-image classification accuracy achieved is **92.78%**

		Predicted class					
		Normal (%)	CLD (%)	CON (%)	PTX (%)	RDS (%)	TTN (%)
True Class	Normal	**98.33**	0	0	0.83	0	0.83
	CLD	0	**100**	0	0	0	0
	CON	0	0	**95.83**	0	4.17	0
	PTX	9.17	0	0.83	**76.67**	0	13.33
	RDS	0	0	4.17	0	**92.5**	3.33
	TTN	3.33	0	0	3.33	0	**93.33**

The key metrics, overall classification accuracy, the weighted F1-score, and the classification accuracy for each class were shown in bold

The weighted F1-score is **0**.**927**

**Table 4:** Classification confusion matrix for results with LOO CV on the IMBALANCED data set using top 15 DTCWT features and three clinical features. The overall per-image classification accuracy achieved is **74.39%**

		Predicted class					
		Normal (%)	CLD (%)	CON (%)	PTX (%)	RDS (%)	TTN (%)
True Class	Normal	**17.84**	0	0	7.03	0	75.14
	CLD	0	**100**	0	0	0	0
	CON	0	0	**99.02**	0	0.98	0
	PTX	8.57	0	12.38	**17.14**	25.71	36.19
	RDS	2.86	0	8.98	5.31	**57.55**	25.31
	TTN	4.53	0	0	2.26	2.83	**90.38**

The key metrics, overall classification accuracy, the weighted F1-score, and the classification accuracy for each class were shown in bold

The weighted F1-score is **0**.**740**

**Table 5:** Classification confusion matrix for results with LOSO CV on the BALANCED data set using top 15 DTCWT features and three clinical features. The overall per-subject classification accuracy achieved is **75%**

		Predicted class					
		Normal (%)	CLD (%)	CON (%)	PTX (%)	RDS (%)	TTN (%)
True class	Normal	**93.33**	0	0	1.67	0	5
	CLD	0	**100**	0	0	0	0
	CON	0	0	**78.33**	0	21.67	0
	PTX	11.67	0	2.5	**40.83**	5.83	39.17
	RDS	0	0	28.33	0	**52.5**	19.17
	TTN	9.17	0	0	5.83	0	**85**

The key metrics, overall classification accuracy, the weighted F1-score, and the classification accuracy for each class were shown in bold

The weighted F1-score is **0**.**713**

**Table 6:** Classification confusion matrix for results with LOSO CV on the IMBALANCED data set using top 15 DTCWT features and three clinical features. The overall per-subject classification accuracy achieved is **63.48%**

		Predicted class					
		Normal (%)	CLD (%)	CON (%)	PTX (%)	RDS (%)	TTN (%)
True Class	Normal	**4.86**	0	0	11.89	0	83.24
	CLD	0	**100**	0	0	0	0
	CON	0	0	**96.39**	0	3.61	0
	PTX	23.81	0	15.24	**3.81**	22.86	34.29
	RDS	5.71	0	17.96	6.12	**39.59**	30.61
	TTN	13.96	0	0	5.09	5.47	**75.47**

The key metrics, overall classification accuracy, the weighted F1-score, and the classification accuracy for each class were shown in bold

The weighted F1-score is **0**.**606**

**Fig. 4:**
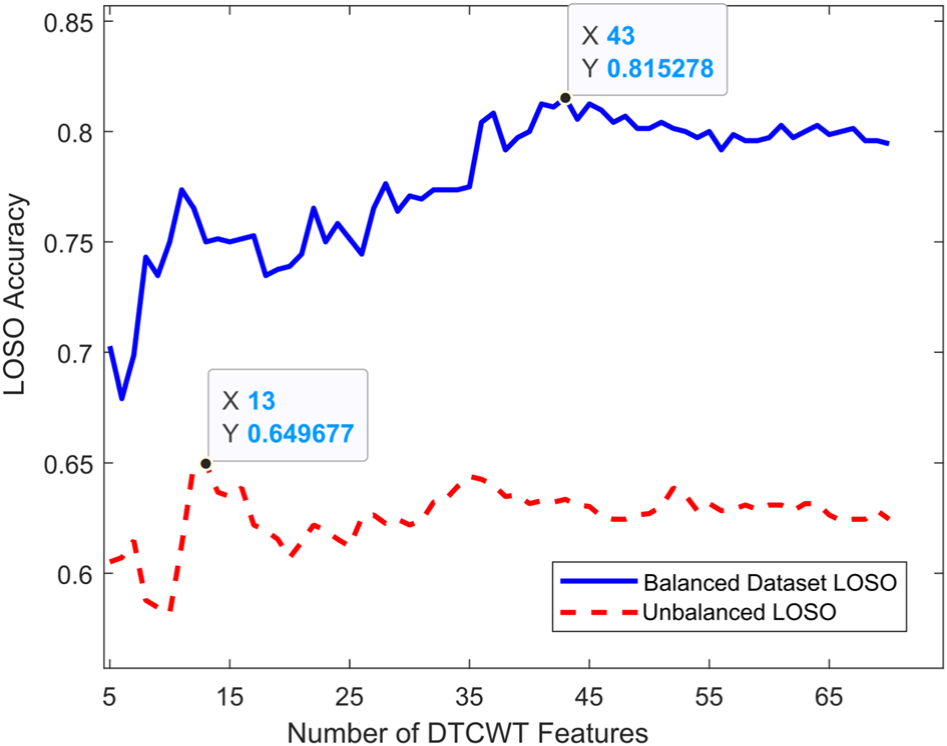
LOSO accuracies for balanced and imbalanced data set with increased number of DTCWT features

Moving on to the LOSO results in Tables 5 and 6, we see, in general, a reduction as expected in the per-subject classification accuracies in comparison with the per-image classification accuracies in Tables 3 and 4. This could be explained for two reasons. First, we have removed any subject bias that may have influenced the results using LOO CV with images. Secondly, LOSO CV reduces the amount of condition-specific data the model is trained on for the patient in the testing set, which affects the model’s accuracy and could lead to a decrease in accuracy. In addition, for the imbalanced data set case, the imbalanced and 1/3rd presence of the TTN might have amplified the difficulty level of classifying the groups. Lastly, the reduced feature dimension might not have enough discriminative ability to separate the groups well. The weighted F1 score was 0.713 on the balanced data set and 0.606 on the imbalanced data set.

Additionally, to examine whether additional DTCWT features could further improve our classification accuracy, we reiteratively implemented our pattern classification method with LOSO while gradually increasing the number of DTCWT features. We show in Fig. 4 that a maximum classification accuracy of 81.53% for a balanced data set could be achieved if the first 43 DTCWT features were included in our LDA model.

## Discussion

Our work attempts to automate the detection of LUS morphologies to diagnose lung pathologies among neonates in developing countries. Below are the primary findings of our work.

- All six typical neonatal lung pathologies observed in the NICU could be classified using line and textural features of POC–LUS images and clinical features.
- Features extracted from LUS images using 2D DTCWT and clinical features demonstrate the potential to accurately separate the six most common neonatal lung pathologies.

Previous works have demonstrated wavelet transform and its variants to detect line and texture patterns from LUS images to classify and assess lung pathologies. Jiao et al. [8] and Du et al. [10, 11] independently showed that NRM of an infant could be predicted using textural and wavelet features extracted from fetal LUS images. Clinical features, such as GA, gestational diabetes mellitus, and pregnancy complications, could augment the prediction of infant NRM. Bonet-Carne’s team used the same feature types as the aforementioned studies to predict NRM; however, they extracted features from the LUS of neonates [9]. We extend the work of Bonet-Carne et al. by exploring different texture and wavelet features of neonatal LUS images and expanding the classification of neonatal lung pathologies beyond RDS and TTN.

One of the disadvantages of a traditional Discrete Wavelet Transform (DWT) is its inherent spatial shift variance. Thus, features extracted using DWT may not be consistent due to natural spatial shifts that occur during LUS scans. DTCWT minimizes the shift variance from DWT by doubling the sample rate at each level of decomposition [15]. We take advantage of DTCWT’s directional selectivity, great spatiospectral localization, and scale- and shift-invariance to extract morphologies of LUS images and automate the detection of the six most common neonatal lung pathologies.

We also demonstrated high classification accuracy using a simple linear classifier on a balanced data set. While LDA produces only a conservative estimate, we showed that we could achieve 92.78% per image and 75.00% per subject cross-validated classification accuracy via LOO and LOSO, respectively. While more intricate deep-learning classifiers have been used for the classification of lung pathologies from LUS [13, 23–26], we illustrated that we could still achieve great classification accuracy using LDA only. Our classification results using only a linear classifier reflect the robustness of our proposed LUS image and clinical features.

The 3 most commonly selected features for LOO splits on the balanced data set were M2 Mean, M1 GLCM Contrast and M1 GLCM Homogeneity. M2 Mean is the global mean of the 2nd level low passed band. Normal, TTN and PTX have a much lower M2 mean than the other classes. The other 3 conditions, RDS, CLD and CON, have greater M2 Means as they all commonly have large areas of coalescent B-lines leading to a greater mean value in the M2 sub-image. M1 GLCM contrast is the GLCM contrast of the 1st level low passed band. A larger value is due to a greater difference in intensity values among neighbouring pixels. Images with texture patterns that have large intensity differences, such as areas with consolidations, will have greater M1 GLCM contrast values. M1 GLCM Homogeneity is the GLCM homogeneity of the 1st level low passed band. GLCM Homogeneity is a measure of the local homogeneity of an image Images with large areas of similar intensity, such as areas with coalescent B-lines and large black areas (such as the areas between A-lines) will have greater M1 GLCM homogeneity values.

We tested the performance of the algorithm using two different cross-validation methods. The results using LOO CV were generally higher than LOSO CV due to the difference in per-subject and per-image database size biases. The LOO CV results provide an inflated estimate of model performance while LOSO CV reduces the amount of condition-specific data the model is trained on for the patient in the testing set resulting in conservative results.

Since we included only the top fifteen DTCWT with clinical features, we also examined whether additional DTCWT features could further improve our classification accuracy. Therefore, along with our three clinical features, we reiteratively implemented our pattern classification method with LOSO while gradually increasing the number of DTCWT features. We show in Fig. 4 that a maximum classification accuracy of 81.53% for a balanced data set could be achieved if the first 43 DTCWT features were included in our LDA model. Including the first 43 DTCWT features offers an incremental improvement to our previous classification accuracy by 6.5%

We further compared the classification performance of LDA using our proposed set of 2D DTCWT features against our previous work using the balanced and imbalanced data sets [14]. We achieved 85.42% and 72.00% LOO cross-validated classification accuracies on our balanced and imbalanced data sets, respectively, using recurrence features only: lower than classification accuracies when DTCWT features are used.

We tested the performance of the algorithm on two different data sets, a balanced data set and an imbalanced data set. The results on the balanced data set were overall better than the imbalanced data set. The imbalanced data set was biased toward TTN based on the distribution of the training set.

In our future research, we intend to explore dynamic features that detect pleural sliding movement, which could further aid in distinguishing these conditions. Notably, PTX is the only condition without a pleural sliding sign, making its absence a clear indicator to rule out both Normal and TTN. Additionally, the differentiation between Normal and TTN becomes straightforward with the presence of more than 3 B-lines, favoring TTN, and their absence favoring the normal lung pattern. This provides an immediate indicator to rule out PTX.

Our work introduces a multi-class classification approach to aid in the automated diagnosis of common neonatal lung pathologies based on LUS morphologies. While our study shares some similarities with the previous work by Veeramani et al. where, in addition to PTX, TTN, and RDS, lung pathologies such as Meconium Aspiration Syndrome, bronchiolitis, pneumonia, and lung cancer were classified [12], our work uses an expanded data set curated by well-trained clinicians to match traditional LUS morphologies of neonatal lung pathologies observed in practice. We also extend the work by Bassiouny et al. [13] by performing direct pathology classification and extracting meaningful LUS morphologies of neonatal lung pathologies. Including relevant clinical features also enabled us to distinguish specific lung pathologies that would be otherwise challenging to classify using only LUS morphologies. Finally, our work forgoes segmenting key lung areas for feature extraction as in [8, 10, 18, 23, 27, 28]. Rather, we separated each image into its top and bottom halves to localize pleural lines and image features associated with the amount of air in the lungs, respectively. Our proposed framework reduces the overall complexity of neonatal lung pathology diagnosis using POC-US images. Moreover, our method alleviates the need for expert input via manual or semi-automatic supervision of image processing from POC-US especially at healthcare institutions that lack well-trained physicians.

### Limitations

Our feature selection method could be improved using advanced machine learning methods, which is outside our current work’s scope. We chose the top fifteen DTCWT features to create training sets that are 10% to 15% of our sample which produced a conservative performance without overfitting, as we demonstrated.

We also recognize that deep-learning-based classifiers could improve lung pathologies’ classification accuracy from LUS images as demonstrated by previous works [23, 24, 26]. Nonetheless, we demonstrated that a simple linear classifier could provide a high yet conservative cross-validated classification accuracy. A simple linear classifier was chosen over the complex non-linear classifiers to emphasize extracting meaningful features relevant to clinical markers and keep the outcomes conservative and realistic. Furthermore, the performance of linear classifiers could be attributed to the close relationship between features used for classification and observed phenomena, such as LUS morphologies of neonatal lung pathologies. In contrast, larger data sets with complex relationships could benefit more from deep-learning-based classifiers.

Finally, we excluded motion-related LUS morphologies for detecting PTX due to the inherent characteristics of neonatal lungs. Summers et al. showed that LUS images associated with PTX have unique motion artifacts in adults. However, PTX in neonates could still be classified without motion-related LUS morphologies since neonates with PTX have air between their pleural linings, natural dampers and non-echoic. Therefore, the lack of LUS morphology for fluids directly indicates PTX in neonates without the need for motion-related artifacts.

## Conclusions

We demonstrated that DTCWT features of LUS images and clinical features could be used to classify typical LUS pathologies among neonates. With an expanded data set, a simple linear classifier could achieve conservative yet high cross-validated classification accuracy. Classifier performance could be attributed to capturing clinically relevant morphologies and patterns via DTCWT features as observed in practice. In conjunction with clinical features, line and textural features of POC–LUS images allow for the classification of all six common neonatal lung pathologies observed in the NICU. Using 2D DTCWT for extracting features from LUS images, combined with clinical characteristics, shows promise in effectively distinguishing the six prevalent neonatal lung pathologies. Automated classification of LUS images could serve as a point-of-care screening tool to identify lung pathologies among neonates. Our future work will focus on extracting dynamic features for detecting lung sliding from post-processed LUS videos for classifying PTX and using advanced machine learning and data fusion techniques. Our current work could aid in automating diagnostic systems in healthcare institutions that lack well-trained clinicians to interpret neonatal LUS images. Better treatment strategies could be developed with an early and accurate diagnosis of lung pathologies among neonates and aid in decreasing neonatal morbidities and mortalities in developing countries.

## Methods

This section presents the methods and processes for obtaining the DTCWT features and performing pattern classifications.

### Data set

**Fig. 5:**
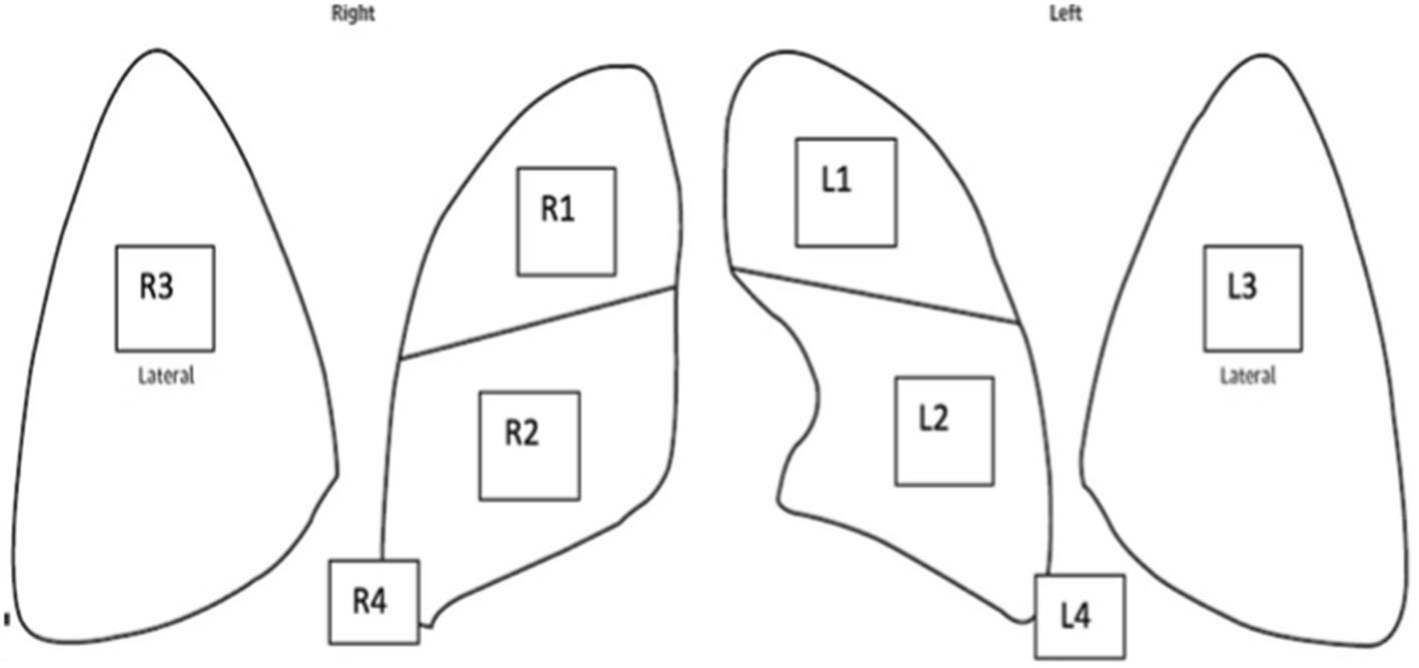
The 6 standard lung regions scanned during lung ultrasound (L1, L2, L3, R1, R2, R3). This is a standard clinical method

Our clinical collaborator and expert in neonatal LUS (AM) from a Canadian tertiary neonatal intensive care unit in Mount Sinai Hospital (MSH) acquired all of the LUS scan videos. All scans were performed using a portable ultrasound machine (Z.One PRO Ultrasound System, Mindray North America, CA, USA) with a high-resolution linear probe (L20-5) to improve image quality and adequate penetration to help with lung image acquisition. Research Ethics Board approvals and data-sharing agreements were obtained from Toronto Metropolitan University and MSH. A breakdown of the number of patients, videos, and images for each condition are shown in Table 7. Two data sets were used for this work: an imbalanced data set (class imbalanced) and a balanced data set. The imbalanced data set comprised all of the data sets acquired by clinical collaborators. The imbalanced data set contains data distribution (i.e. number of patients per group) as collected by and as available in the NICU of the collaborating hospital. This distribution of the groups within this data set is not a true reflection of a real-world distribution, so to avoid the bias due to an uneven number of patients per group we also created a balanced data set with an equal number of patients in each of the groups. Our clinical collaborators selected the balanced data set of four patients per condition to create a data set that does not have a large class imbalance biased towards TTN, which comprises more than a third of the data set in terms of the number of images. This was done to train a model free of bias based on the distribution of the training set. Our pre-processing step was done to avoid the considerable similarity between neighboring frames that would bias our classification model. The data set was created using five frames from each video our clinical collaborators acquired. The imbalanced data set contained six or more videos for most of the patients in the data set. For most patients, one or more videos for commonly imaged areas of the lungs (R1, R2, R3, L1, L2, and L3, as shown in Fig. 5) were included in the data set. R4 and L4 regions are imaged for specific conditions (pleural effusion) but not for any conditions in the data set. For patients with PTX, as it happens in a specific area of the lung pleura and rarely in both lungs, only lung areas with pneumothorax were included, so these patients usually had less than six videos. Five frames were taken simultaneously in the video for the imbalanced data set unless a particular frame was unclear. The second data set, the balanced data set, consisted of only four patients per condition. Six videos, one for each lung region, were taken for each patient, and five frames taken at equal intervals were used from each video, as shown in Table 8. For 2 of the PTX patients, only 3 and 4 videos were available, so 10 and 7 or 8 frames were taken from those videos to have 30 images per patient. Our clinical collaborators determined the patients and videos for the balanced data set. The balanced data set was created to test the performance of the DTCWT features without any effects of bias stemming from a class imbalanced data set.

**Table 7:** Imbalanced data set overview

	Patients	Videos	Images
Normal	6	37	185
CLD	6	36	180
CON	7	61	305
PTX	5	21	105
RDS	7	49	245
TTN	11	106	530
**Total**	**42**	**310**	**1550**

**Table 8:** Balanced data set overview

	Patients	Videos	Images
Normal	4	24	120
PTX	4	19	120
TTN	4	24	120
CLD	4	24	120
RDS	4	24	120
CON	4	24	120
**Total**	**24**	**139**	**720**

### Preprocessing

**Fig. 6:**
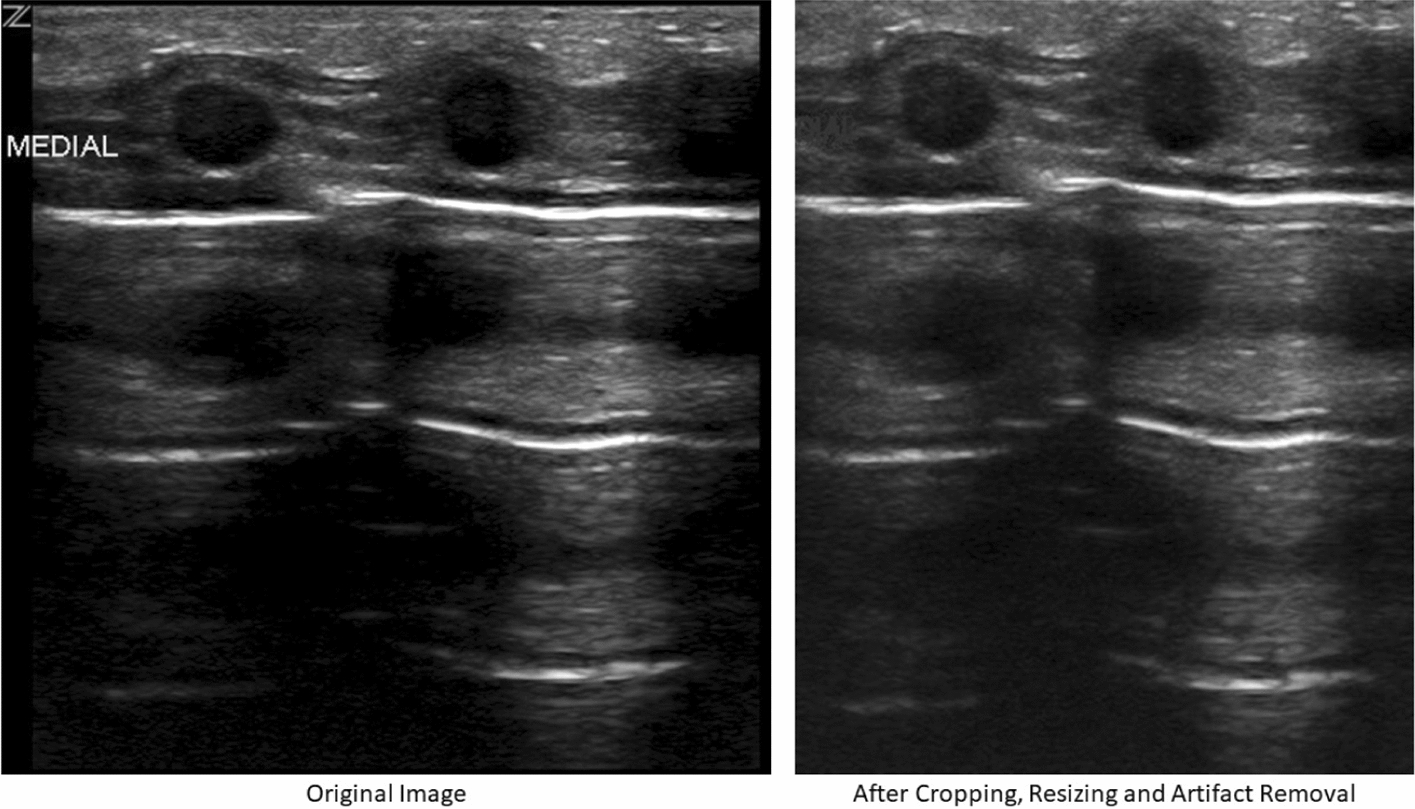
Artifact removal and image normalization

Preprocessing of LUS images consisted of two operations: artifact removal and normalization. LUS videos have regions with patient information and other overlaid artifacts close to the image’s max intensity. LUS video artifacts remain at the exact x–y coordinates throughout a video, so they were removed semi-automatically by selecting a region of interest (ROI), including the artifacts for all frames of an LUS video. LUS artifacts were removed by selecting pixels in the ROI with intensities greater than 50% of the maximum intensity. The 8-pixel connected neighbors were also selected. All the selected pixels were replaced with median intensity for the ROI. For normalization, LUS images were resized to [520 420]. Also, ten columns/rows of pixels were removed from each side of the image, resizing to [500 400]. The image resizing steps were performed to create a uniform image size for the images in the data set and to remove any non-ultrasound pixels from the edges of some of the images. These steps were taken to remove the high-intensity artifacts that can easily be picked up by some of the sub-images and normalize the images for DTCWT decomposition. Finally, LUS images were cropped to remove unimportant parts and artifacts that appeared at the edges of the video. An example of the preprocessing steps is provided in Fig. 6.

### Time-frequency/time-scale transformation

The LUS morphologies combine spatially localized information, texture patterns, and small oscillations. These patterns can be characterized as belonging to different spatial frequencies and relative locations, so 2D time-frequency/time-scale transformations may be able to capture these patterns, making extracting features that can quantify them easier.

#### DWT

The 2-D discrete wavelet transform (DWT) can be used to analyze an image and isolate these different frequencies using different scales. The 2-D discrete wavelet transform uses basis functions to decompose various scaled versions of the input image [29, 30]. The 2-D DWT is implemented by using 1D wavelet and scaling functions [29]. The 1D DWT of a signal can be defined as

1 $$\begin{aligned} x[n]=\sum _{k}{a_{J,k}2^{-J/2}\phi (2^{-J}n-k)}+\sum _{j,k}{d_{j,k}2^{-j/2}\psi (2^{-j}n-k)} \end{aligned}$$

Where $a_{J,k}$ is the approximation coefficients and $d_{j,k}$ is the detail coefficients at octave decomposition levels *j*. $\psi (n)$ is the orthonormal wavelet function, $\phi (n)$ is the scaling function and *k* is the translation parameter.

**Fig. 7:**
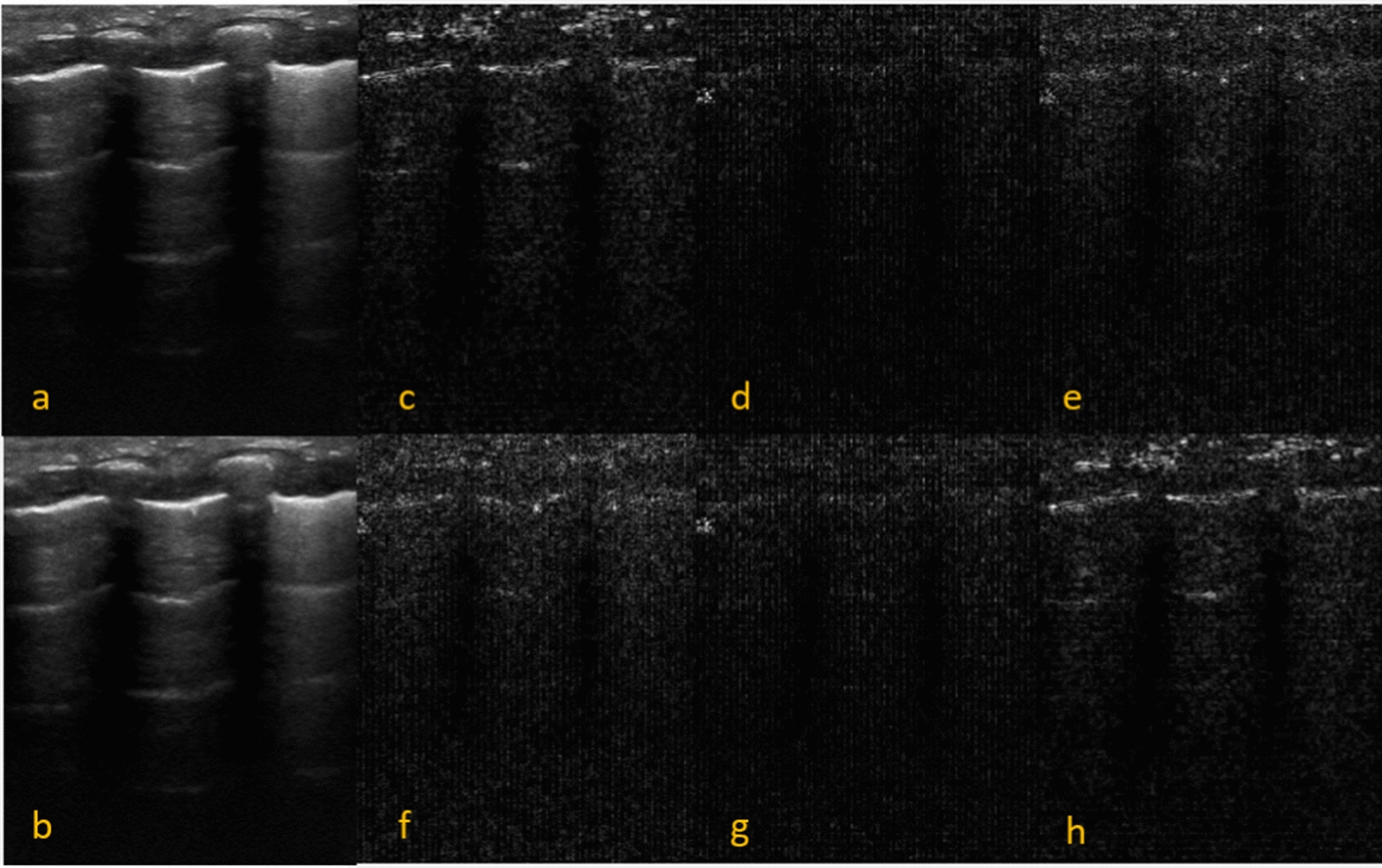
One level of DTCWT decomposition.** a**: Image of Patient with TTN,** b**: Low-Passed Band: smoothed image** c**: Subimage at +15$^{\circ }$,** d**: Subimage at +45$^{\circ }$,** e**: Subimage at 75$^{\circ }$,**f**: Subimage at -75$^{\circ }$,** g**: Subimage at -45$^{\circ }$,** h**: Subimage at -15$^{\circ }$)

Using the 2D-DWT an image $I(k_1,k_2)$ can be decomposed as follows[31]:

2 $$\begin{aligned} \sum _{k_{1}=0}^{N}\sum _{k_{2}=0}^{M}I(k_{1},k_{2})f^{d}_{j}(k_{1}-n_{1},k_{2}-n_{2})= \left\{ \begin{array}{ll} A_{j} &{} if\ d=0 \\ Dh_{j} &{} if\ d=1 \\ Dv_{j} &{} if\ d=2 \\ Dd_{j} &{} if\ d=3 \\ \end{array} \right. \end{aligned}$$

Where *N* and *M* are the row and column numbers. $A_{j}$ are the approximation coefficients or approximation sub-images and $Dh_{j}$, $Dv_{j}$ and $Dd_{j}$ are the detail coefficients or detail sub-images for every level of decomposition j. The function $f^{d}_{j}$ is defined as:

3 $$\begin{aligned} f^{d}_{j}= \left\{ \begin{array}{ll} \phi _{j}^*(n_{1})\phi _{j}^*(n_{2}) &{} d=0 \\ \phi _{j}^*(n_{1})\psi _{j}^*(n_{2}) &{} d=1 \\ \psi _{j}^*(n_{1})\phi _{j}^*(n_{2}) &{} d=2 \\ \psi _{j}^*(n_{1})\psi _{j}^*(n_{2}) &{} d=3 \\ \end{array} \right. \end{aligned}$$

However, an issue with the DWT is that it is shift-variant. This means that if the morphologies in the LUS images were translated, the DWT would generate a different set of DWT coefficients.

#### DTCWT

DTCWT-type approaches can be used as it is nearly shift-invariant. Also, DTCWT has good directional selectivity and perfect reconstruction [32]. A 2D time-frequency/time-scale transformation with shift-variance, good directional selectivity and nearly perfect reconstruction may capture spatially localized information, texture patterns, and small oscillations [33]. Isolating these patterns makes it easier to quantify these patterns using image features. The LUS morphologies are a combination of these patterns so that these patterns can provide clues about the morphologies in the image. A 2D DTCWT uses two separate decomposition trees to calculate the complex transform of an image. One of the trees is used to calculate the real parts of the complex coefficients, and the other is used to calculate the imaginary parts of the complex coefficients [15]. The DTCWT is implemented by using two separate two-channel filter banks. Approximate shift invariance was achieved by doubling the sampling rate at each tree level by using two trees [15]. DTCWT decomposition of an image uses a complex scaling function and six wavelet functions [15, 34]. The operational cost of the DTCWT is 4O(MN) where M and N are the length and width of the image [35, 36]. After the DTWCT decomposition statistical, Grey-Level Co-occurrence Matrix (GLCM), Grey-Level Run Length Matrix (GLRLM) and Local Binary Pattern (LBP) features are extracted from the decomposition subimages produced by the DTCWT. The DTCWT results in a low-passed version of the image at each decomposition level and six high-frequency sub-images at each decomposition level corresponding to the six wavelet functions oriented at angles $\alpha$=(±15$^{\circ }$,±45$^{\circ }$,±75$^{\circ }$). The near-symmetric biorthogonal filter pair with lengths 5 (scaling filter) and 7 (wavelet filter) was used for the mother wavelet and the images were decomposed to 5 levels. An example of the 1st level decomposition of a TTN image is shown in Fig. 7.

The decomposition of an image $I(k_1,k_2)$ can be performed by using a complex scaling function and six complex wavelet functions as follows [37]:

4 $$\begin{aligned} I(k_1,k_2)=\sum \limits _{l\in Z^{2}} {A_{j_{0},l} \phi _{j_{0},l} (k_1,k_2)}+ \sum \limits _{g\in \alpha } {\sum \limits _{j=1}^{j_{0} } {\sum \limits _{l\in Z^{2}} {D_{j,l}^{g} } } } \psi _{j,l}^{g} (k_1,k_2)\quad \end{aligned}$$

Where $j_0$ is the number of decomposition level, $A_{j0,l}$ and $D_{j,l}^{g}$ are scaling coefficients and wavelet coefficients respectively. $\phi _{j_{0},l}(k_1,k_2)$ represents the scaling function and $\psi _{j,l}^g(k_1,k_2)$ represents the six wavelet functions [37].

### Feature extraction

We extracted features from the top and bottom half of the DTCWT sub-images. The rationale is the top half of the images will have features related to the pleural line and the bottom half will have features related to B-lines and A-lines. We used statistical features directly on the image to extract features that can describe the intensity distribution of the pixels, grey-level co-occurrence features, and rotation-invariant uniform LBP features. We used grey-level run length matrix features to extract features that measure the morphology in the image. A combination of textural and morphological features was selected to obtain a complementary feature set that should produce a more robust classification model [38]. Statistical features were extracted from the pixel intensities of the images [39].

#### Statistical features

The statistical features below were chosen as they are commonly used as global features in medical image analysis.

5 $$\begin{aligned} Mean= & {} \frac{\sum _{i=1}^{n} \sum _{j=1}^{m} (I_{x,y})}{m\cdot n} \end{aligned}$$

6 $$\begin{aligned} SD= & {} \frac{\sqrt{\sum _{i=1}^{n} \sum _{j=1}^{m} (I_{x,y}-Mean)^2}}{m\cdot n} \end{aligned}$$

7 $$\begin{aligned} Skewness= & {} \frac{\sum _{i=1}^{n} \sum _{j=1}^{m} (I_{x,y}-Mean)^3}{m\cdot n\cdot SD^3} \end{aligned}$$

8 $$\begin{aligned} Kurtosis= & {} \frac{\sum _{i=1}^{n} \sum _{j=1}^{m} (I_{x,y}-Mean)^4}{m\cdot n\cdot SD^4} -3 \end{aligned}$$

9 $$\begin{aligned} Entropy=- & {} \sum _{i=1}^{n} \sum _{j=1}^{m} I_{x,y}log_{2}(I_{x,y}) \end{aligned}$$

Where *n* is the number of rows in the image, *m* is the number of columns in the image, *i* is the row number, *j* is the column number and *I* is the image.

#### GLCM features

The grey-level co-occurrence matrix is calculated by determining how often pairs of pixels with specific values and in a specified spatial relationship occur in an image. Then, statistical measures are extracted from the GLCM [40]. To calculate the GLCM we first quantized the image to 8 levels, generating an 8x8 GLCM, and chose 6 offsets, [0 1; 1 0; 0 2; 2 0; 1 1; 2 2;], to generate 6 different GLCMs. Our previous work shows that quantization to 8 intensity levels accentuates the differences around the LUS morphologies, increasing the ability of the features to pick up these morphologies [14]. Element (*i*, *j*) of the gray level co-occurrence matrix represents the number of occasions a pixel with intensity *i* is adjacent to a pixel with intensity *j* in the LUS image. The GLCM stores the co-occurring values representing the distance and angular spatial relationship of pixels in a structured matrix. We then extracted 5 features from the GLCM [41].

10 $$\begin{aligned} Contrast= & {} \sum _{i=1}^{n} \sum _{j=1}^{m}(i-j)^2 (G_{i,j}) \end{aligned}$$

11 $$\begin{aligned} Correlation= & {} \sum _{i=1}^{n} \sum _{j=1}^{m} \frac{(i-u)(j-u)(G_{i,j})}{\sigma ^2} \end{aligned}$$

12 $$\begin{aligned} Energy= & {} \sum _{i=1}^{n} \sum _{j=1}^{m} {(G_{i,j})^2} \end{aligned}$$

13 $$\begin{aligned} Homogeniety= & {} \sum _{i=1}^{n} \sum _{j=1}^{m} \frac{(G_{i,j})}{1+(i-j)^2} \end{aligned}$$

14 $$\begin{aligned} Entropy= & {} -\sum _{i=1}^{n} \sum _{j=1}^{m} (G_{i,j}log_2(G_{i,j}) \end{aligned}$$

Where *i* is the row number of the GLCM, *j* is the column number of the GLCM and *G* is the GLCM. Where *u* is the mean pixel intensity in the quantized image and $\sigma ^2$ is the pixel intensity variance in the quantized image.

#### GLRLM features

The grey-level run length matrix stores run lengths based on the grey-level value and length of the run. A grey-level run is a set of pixels with the same grey-level value, which are consecutive and collinearly distributed in some given direction [42]. We calculated the 4 GLRLM for each of the 2 ROIs in the image using 4 directions 0$^{\circ }$, 45$^{\circ }$, 90$^{\circ }$ and 135$^{\circ }$. Then extracted 11 features from the GLRLM. The equations are shown in Table 9 [43, 44].

**Table 9:** GLRLM equations

$\displaystyle LRE=\frac{\sum _{i=1}^{n} \sum _{j=1}^{m}j^2 (R_{i,j})}{\sum _{i=1}^{n} \sum _{j=1}^{m} R_{i,j}} \qquad {(14)}$	$\displaystyle SRE=\frac{\sum _{i=1}^{n} \sum _{j=1}^{m} \frac{(R_{i,j})}{j^2}}{\sum _{i=1}^{n} \sum _{j=1}^{m} R_{i,j}} \quad {(15)}$
$\displaystyle GLN=\frac{\sum _{i=1}^{n} (\sum _{j=1}^{m} (R_{i,j})^2)}{\sum _{i=1}^{n} \sum _{j=1}^{m} R_{i,j}} \quad {(16)}$	$\displaystyle RLN=\frac{\sum _{j=1}^{m} (\sum _{i=1}^{n} (R_{i,j})^2)}{\sum _{i=1}^{n} \sum _{j=1}^{m} R_{i,j}} \quad {(17)}$
$\displaystyle RP=\frac{\sum _{i=1}^{n} (\sum _{j=1}^{m} R_{i,j})}{N} \qquad {(18)}$	$\displaystyle LGRE=\frac{\sum _{i=1}^{n} \sum _{j=1}^{m} \frac{(R_{i,j})}{i^2}}{\sum _{i=1}^{n} \sum _{j=1}^{m} R_{i,j}} \quad {(19)}$
$\displaystyle HGRE=\frac{\sum _{i=1}^{n} \sum _{j=1}^{m}i^2 (R_{i,j})}{\sum _{i=1}^{n} \sum _{j=1}^{m} R_{i,j}} \qquad {(20)}$	$\displaystyle SRLGE=\frac{\sum _{i=1}^{n} \sum _{j=1}^{m} \frac{(R_{i,j})}{i^2\cdot j^2}}{\sum _{i=1}^{n} \sum _{j=1}^{m} R_{i,j}} \quad {(21)}$
$\displaystyle SRHGE=\frac{\sum _{i=1}^{n} \sum _{j=1}^{m} \frac{i^2(R_{i,j})}{j^2}}{\sum _{i=1}^{n} \sum _{j=1}^{m} R_{i,j}} \qquad {(22)}$	$\displaystyle LRLGE=\frac{\sum _{i=1}^{n} \sum _{j=1}^{m} \frac{j^2(R_{i,j})}{i^2}}{\sum _{i=1}^{n} \sum _{j=1}^{m} R_{i,j}} \quad {(23)}$
$$\displaystyle \hspace{27.5 mm} LRHGE=\frac{\sum _{i=1}^{n} \sum _{j=1}^{m} i^2\cdot j^2 (R_{i,j})}{\sum _{i=1}^{n} \sum _{j=1}^{m} R_{i,j}} \quad {(24)}$$	

$^{*}$In the above GLRLM feature equations, *i* is the grey level, *j* is the run length and *R* is the GLRLM. The definitions for the features are provided in the Abbreviation section

#### LBP features

The LBP histogram is used to store LBP pixel labels for the image. The pixel labels are calculated by thresholding the 3x3-neighborhood of each pixel with the center value and considering the result as a binary number [45]. We used the rotation-invariant LBP, which resulted in 10 bins. First, only uniform LBPs, with a maximum of two circular 0-1 or 1-0 transitions, are considered unique patterns. All non-uniform patterns are stored in one bin. Then, in the rotation-invariant version, LBP patterns that result in the same value when rotated are stored in the same bin. The values of the bins were used as the feature set.

#### Clinical features

As used in previous works and our initial work [8, 9, 14], we included three clinical features (Gestational Age (GA), Cumulative Gestational Age at the Time of Scan (CGATS) and Days of Life (DOL)) in our models as they are essential in differentiating cases with CLD versus RDS as their features in LUS may overlap. Further, our clinical collaborators affirmed that the LUS image morphologies could not separate the conditions. Without these features, a meaningful clinical diagnosis of the conditions is impossible or extremely difficult only using LUS images. While these are essential features in a classification sense, it is critical to note that the clinical features cannot help differentiate other conditions such as PTX and normal lung. For example, Normal and PTX are unrelated to the clinical features and can occur in neonates regardless of gestation age, days of life, and age at the time of the scan. The clinical features can only separate the conditions meaningfully and play a supportive role when LUS information is available.

### Pattern classification

In this work, we used a simple Linear Discriminant Analysis (LDA) based classifier to classify the 6 LUS conditions using the features extracted from the LUS images. A simple linear classifier, such as an LDA, was chosen over the complex non-linear classifiers to emphasize extracting meaningful LUS features relevant to clinical markers and keep the outcomes conservative and realistic. More importantly, LDA is less prone to overfitting when compared to more complex models [46], which is very important when dealing with a small data set. While performing classification on the balanced data set, the models were trained based on equal prior probabilities between the groups. For the imbalanced data set, we used prior probabilities based on group size. A simple linear classifier was chosen over the complex non-linear classifiers to emphasize extracting meaningful features relevant to clinical markers and keep the outcomes conservative and realistic. Similarly, we employed a univariate feature selection approach utilizing Chi-square tests [47], as we needed a feature selection method with low computational complexity for performing feature selection within the cross-validation loop. In the Chi-square feature selection process, an individual Chi-square test is performed for each feature and the class labels. A small p value indicates that the corresponding feature depends on the class and is ranked accordingly as an essential feature. Chi-square feature selection selects the features with the lowest p values as the top features [48]. For the results given in Tables 3, 4, 5 and 6, the features with the 15 lowest p values were chosen. Feature selection was performed within the cross-validation loops to avoid data leakage.

In terms of cross-validation, we tested the performance using two forms of cross-validation. First, we used leave-one-out cross-validation (LOO CV), a form of cross-validation where only one image is used as the testing set, and the model is trained on the rest of the images. This is repeated so that each image is used as a testing set. The average accuracy of all these runs then results in per-image classification performance. We also used leave-one-subject-out cross-validation (LOSO CV). In this form of cross-validation, all images belonging to a subject are used as the testing set and the rest of the images as the training set. This is repeated until each subject has served in the testing set. The average accuracy of all these runs then results in per-subject classification performance. The motivation behind the LOSO cross-validation is to avoid biasing the model due to the similarity between images from the same patient. LOO CV was used to estimate the system’s performance when used to make predictions on data not used to train the model. LOSO CV was used to estimate the system’s performance when used to make predictions on a subject that was not used to train the model. We only selected five images from each video to avoid the significant similarity between frames in the same video for our LOO results. The images were selected at equal intervals throughout the video. If the lung was not imaged in the frame, another frame was selected, as at the beginning or end of a few videos. In the results section, we provide overall classification accuracies, confusion tables and weighted F1-score. The weighted F1-score computes the F1 score for individual classes and then averages using each class’s true label count [49].

## Data Availability

The study uses retrospective neonatal clinical data that is restricted both by the REB and data-sharing policies of the hospital and hence cannot be shared or made available to the public.

## Abbreviations

CGATS: Cumulative gestational age at the time of scan
CLD: Chronic lung disease
CON: Consolidations
DOL: Days of Life
DTCWT: Dual-tree Complex wavelet transform
DWT: Discrete wavelet transform
EEG: Electroencephalography
FRCNN: Faster region-based convolutional neural network
GA: Gestational age
GDM: Gestational diabetes mellitus
GLCM: Grey-level co-occurrence matrix
GLN: Grey level non-uniformity
GLRLM: Grey-level run length matrix
HGRE: High grey-level run emphasis
LBP: Local binary pattern
LDA: Linear discriminant analysis
LGRE: Long run high grey-level emphasis
LRE: Long run emphasis
LRLGE: Long run low grey-level emphasis
LRHGE: Long run high grey-level emphasis
LOO CV: Leave-one-out cross-validation
LOSO CV: Leave-one-subject-out cross-validation
LUS: Lung ultrasound
MR: Magnetic resonance
MSH: Mount Sinai hospital
NICU: Linear binary pattern
NRM: Neonatal intensive care unit
NSERC: Natural Sciences and Engineering Research Council of Canada
POC-LUS: Point-of-care lung ultrasound
POC-US: Point-of-care ultrasound
PTX: Pneumothorax
RDS: Respiratory distress syndrome
REB: Research ethics board
ROI: Region of interest
RP: Run percentage
RLN: Run length non-uniformity
SRE: Short run emphasis
SRHGE: Short run high grey-level emphasis
SRLGE: Short run low grey-level emphasis
TTN: Transient tachypnea of the newborn
